# Managing inadequate response to initial anti-TNF therapy in rheumatoid arthritis: optimising treatment outcomes

**DOI:** 10.1177/1759720X221114101

**Published:** 2022-08-16

**Authors:** Peter C. Taylor, Marco Matucci Cerinic, Rieke Alten, Jérôme Avouac, Rene Westhovens

**Affiliations:** Botnar Research Centre, Nuffield Department of Orthopaedics, Rheumatology and Musculoskeletal Sciences, University of Oxford, Old Rd, Headington, Oxford OX3 7LD, UK; Department of Experimental and Clinical Medicine, University of Florence, Florence, Italy; Unit of Immunology, Rheumatology, Allergy and Rare Diseases (UnIRAR), IRCCS San Raffaele Hospital, Milan, Italy; Department of Internal Medicine, Rheumatology, Clinical Immunology and Osteology, Schlosspark-Klinik University Medicine Berlin, Berlin, Germany; AP-HP Centre, Université de Paris, Hôpital Cochin, Service de Rhumatologie, Paris, France; Skeletal Biology and Engineering Research Center, Department of Development and Regeneration and Division of Rheumatology, KU Leuven, Leuven, Belgium

**Keywords:** anti-TNFs, biologics, inadequate response, rheumatoid arthritis

## Abstract

Anti-tumour necrosis factors (anti-TNFs) are established as first-line biological therapy for rheumatoid arthritis (RA) with over two decades of accumulated clinical experience. Anti-TNFs have well established efficacy/safety profiles along with additional benefits on various comorbidities. However, up to 40% of patients may respond inadequately to an initial anti-TNF treatment because of primary non-response, loss of response, or intolerance. Following inadequate response (IR) to anti-TNF treatment, clinicians can consider switching to an alternative anti-TNF (cycling) or to another class of targeted drug with a different mechanism of action, such as Janus kinase inhibitors, interleukin-6 receptor blockers, B-cell depletion agents, and co-stimulation inhibitors (swapping). While European League Against Rheumatism recommendations for pharmacotherapeutic management of RA, published in 2020, are widely regarded as helpful guides to clinical practice, they do not provide any clear recommendations on therapeutic choices following an IR to first-line anti-TNF. This suggests that both cycling and swapping treatment strategies are of equal value, but that the treating physician must take the patient’s individual characteristics into account. This article considers which patient characteristics influence clinical decision-making processes, including the reason for treatment failure, previous therapies, comorbidities, extra-articular manifestations, pregnancy, patient preference and cost-effectiveness, and what evidence is available to support decisions made by the physician.

## Introduction

Rheumatoid arthritis (RA) is a chronic, systemic, inflammatory disorder.^1^ The primary goals of RA treatment are to reduce the signs and symptoms of disease, prevent progression of joint damage and improve patients’ physical function, thereby enhancing their quality of life (QoL). The introduction of biologic disease-modifying anti-rheumatic drugs (bDMARDs) and targeted synthetic DMARDs (tsDMARDs) has broadened the therapeutic options for patients with RA (Table 1). Subsequently, anti-tumour necrosis factor (anti-TNF) therapies (etanercept, infliximab, adalimumab, certolizumab, and golimumab) have become the leading first-line choice in patients with moderate-to-severe RA. In addition to anti-TNFs, other biologic agents used in the treatment of RA are abatacept (co-stimulation modulator), rituximab (anti-CD20 antibody), the anti-interleukin-6 (IL-6) receptor antibodies, tocilizumab and sarilumab or tsDMARDs, such as the Janus kinase inhibitors (JAKi) (e.g. tofacitinib, baricitinib, filgotinib, and upadacitinib).

**Table 1.** Characteristics of reference products from the different therapeutic classes.^2–15^

Product	Structure/MoA	Route	Date of approval	Approved indications (EU)	Available dosage forms
Anti-TNFs					
Infliximab	mAb	IV	August, 1999	RA, CD (P), UC (P), AS, PsA, Pso	Vial
Etanercept	Fc fusion protein	SC	February, 2000	RA, JIA, AS, PsA, Pso (P)	PFS, pen
Adalimumab	mAb	SC	September, 2003	RA, JIA, AS, PsA, Pso (P), CD (P), Uveitis (P), HS (Ad), UC (P)	PFS, pen
Certolizumab	Fab fragment	SC	October 2009	RA, AS, Pso, PsA	PFS
Golimumab	mAb	SC	October 2009	RA, AS, JIA, PsA, UC	PFS, pen
B-cell depletion					
Rituximab	mAb	IV/SC^a^	June 1998	RA, GPA/MPA, NHL, FL, DLBCL, CLL	Vial
Co-stimulation					
Abatacept	Fc fusion protein	IV/SC	May 2007	RA, PsA, JIA	Vial, PFS, pen
IL-6i					
Tocilizumab	mAb	IV/SC	January 2009	RA, JIA, GCA, CAR-T CRS	Vial, PFS, pen
Sarilumab	mAb	SC	June 2017	RA	PFS, pen
JAKi					
Baricitinib	JAK1/2 inhibitor	Oral	February 2017	RA, AD	Tablet
Tofacitinib	JAK1/3 inhibitor	Oral	March 2017	RA, PsA, UC	Tablet
Upadacitinib	JAK1/2 inhibitor	Oral	December 2019	RA, PsA, AS	Tablet
Filgotinib	JAK1 inhibitor	Oral	September 2020	RA	Tablet

Ad, adult; AS, ankylosing spondylitis; CAR-T, chimeric antigen receptor T-cells; CD, Crohn’s disease; CLL, chronic lymphocytic leukaemia; CRS, cytokine release syndrome; DLBCL, diffuse large B-cell lymphoma; EU, European Union; FL, follicular lymphoma; GCA, giant cell arteritis; GPA, granulomatosis with polyangiitis; HS, hidradenitis suppurativa; IV, intravenous; JAK, Janus kinase; JIA, juvenile idiopathic arthritis; MoA, mechanism of action; mAb, monoclonal antibody; MPA, microscopic polyangiitis; N/A, not applicable; NHL, non-Hodgkin’s lymphoma; P, paediatric; PFS, pre-filled syringe; PsA, psoriatic arthritis; Pso, psoriasis; RA, rheumatoid arthritis; SC, subcutaneous; UC, ulcerative colitis.

a Not for RA.

Remission on treatment is currently the best clinical outcome available to patients living with RA. Most will require lifelong treatment, with subsequent dose reduction representing a viable option for some patients.^1^ The European League Against Rheumatism (EULAR) recommends a treat-to-target strategy aiming for sustained clinical remission (or low disease activity) recommending change of therapy if the target is not reached by 6 months on a given treatment. With the growing availability of similarly effective therapeutic agents in RA, tailoring the treatment to the individual patient to minimise time on suboptimal therapies and also taking into account cost-effectiveness becomes increasingly complicated. At present, there are no readily available biomarkers that reliably inform management decisions in routine clinical care although researchers are making progress in the quest for precision medicine approaches.^16^

EULAR recommendations for RA management following inadequate response (IR) to optimised therapy with conventional synthetic DMARDs (csDMARDs, e.g. methotrexate) in patients with poor prognosis give equal status to bDMARDs and tsDMARDs.^1^ Historically, anti-TNFs have accounted for the majority of first-line biological therapies for RA, which may have increased further due to their increased cost-effectiveness following the introduction of biosimilars.^17,18^ However, 30–40% of patients with RA discontinue anti-TNFs due to primary IR (lack of response identified following initial dosing), secondary IR (reduction in initial response over time) or intolerance;^1,19,20^ each situation necessitates a change in treatment regimen.

From clinical experience, two basic therapeutic approaches may follow after anti-TNF-IR: switching to another anti-TNF (cycling strategy) or to a drug with a different mechanism of action (MoA; swapping strategy). The current EULAR guidelines do not include these definitions nor do they specify the use of any particular therapy following anti-TNF-IR, choosing instead to simply suggest that all contraindications and associated risks of subsequent treatments require careful consideration prior to use.

The aim of this review, based on current peer-reviewed literature and the authors’ own clinical experience, is to supplement healthcare professionals’ current knowledge in everyday clinical practice.

## Inadequate treatment response management strategies

Approximately, 30–40% of patients discontinue anti-TNF treatment over an approximate time frame of 2 years because of primary non-response, secondary non-response or intolerance.^20^ However, the numbers of patients reported to discontinue anti-TNFs may vary according to local practice, differing healthcare systems, and importantly the time period studied. For example, a long-term cohort study reporting on the first severe, long-standing refractory patients treated with infliximab reported a maximum of 20% drug survival after 10 years.^21^ The availability of dose flexibility in this study requires consideration along with approximately 15% of patients being switched to a subcutaneously administered anti-TNF despite being sufficiently controlled at 7 years.^22^ Chatzidionysiou *et al.*^23^ recently reported improved drug survival for all therapeutic options following anti-TNF failure where concomitant csDMARDs were used *versus* no csDMARDs.

Current EULAR guidelines suggest that if a bDMARD, such as an anti-TNF, or tsDMARD has failed, treatment with another bDMARD or a tsDMARD should be considered.^1^ While choosing a second anti-TNF following the failure of a first may sound counterintuitive, a cycling strategy remains common because of physicians’ confidence and familiarity using these drugs, relevant pharmacological differences between products and favourable costs.^24,25^ A number of studies have assessed the efficacy of subsequent therapy in patients with RA who were primary or secondary non-responders to anti-TNFs (Supplemental Appendix Table 1). From these data, it can be seen that a considerable proportion of patients may achieve a clinical response following cycling to an anti-TNF. If a second anti-TNF fails, EULAR guidance recommends that patients are treated with a drug with a different MoA.^1^ The table also shows that these patients will respond when switched to another mode of action. Two head-to-head studiess^16,26^ have indicated that there may be some advantage to switching to an alternate MoA *versus* cycling to another anti-TNF in patients who are non-responders but further data are required and the caveats concerning familiarity, patient individualisation and cost are applicable here too.

Patients appear to be swapping through, and becoming refractory to, the available alternative bDMARD options very quickly.^27^ For example, Kearsley-Fleet *et al.*^27^ reported that between 2001 and 2008, 59% of patients received ⩾ 1 anti-TNF before swapping to a second class of bDMARD, compared with 19% in patients treated from 2011 onwards; the hazard ratio (HR) for developing bDMARD refractory disease was 15 times higher among patients treated from 2011 onwards compared with 2001–2008. Such findings need to be treated with some caution given that fewer bDMARDs were available at that time, resulting in swapping being less common.^27^ In addition, there remains no universally accepted definition of ‘refractory RA’;^28,29^ Kearsley-Fleet *et al.*^27^ defined refractory disease as exposure to ⩾ 3 different classes of bDMARDs, irrespective of the reason for failure to each bDMARD. It has since been suggested that refractory RA could be defined as ‘resistance to multiple therapeutic drugs with different structures and mechanisms of action’^30^ and that refractoriness could be further defined by the presence or absence of inflammation, and/or anti-drug antibodies (ADAs). In our view, refractory implies an overall refractoriness to therapy including initial csDMARDs and to one or more subsequent treatments. It could be argued that the more ambitious treatment targets set over the past two decades have led to an increase in incomplete responses to target, thus leading to physician^31^ and patient dissatisfaction and treatment change. This, in turn, can lead to an increase in refractory disease depending on the definition used. In 2021, EULAR has defined three key criteria for difficult-to-treat RA: (a) treatment according to EULAR recommendations and failure of ⩾ 2 bDMARDs)/tsDMARDs (with different mechanisms of action) after failing csDMARD therapy (unless contraindicated); (b) the presence of ⩾1 of: minimum of moderate disease activity; signs and/or symptoms suggestive of active disease; the inability to taper glucocorticoid treatment; rapid radiographic progression; RA symptoms that are reducing QoL and (c) the management of signs and/or symptoms of RA is perceived to be problematic by the treating rheumatologist and/or the patient.^30^

In addition, the psychological status of the patient can influence how they report their experience in the form of the patient global assessment which, in turn, will impact composite scores of disease activity that include this assessment and thereby increase the likelihood of immunosuppressive treatment escalation with a view to attaining a remission target. Such a treatment change may not be appropriate if the reported symptomatology is not due to an inflammatory cause. Furthermore, in a recent meta-analysis of 11 recent clinical trials in RA, omission of the patient global score from the current, four-variable ACR/EULAR Boolean-based definition of remission^32^ would result in 19% fewer patients undergoing treatment escalation but with minimal adverse consequence in terms of subsequent structural damage progression.^33^ The presence of depression has also been suggested to exacerbate pain and disease activity and decrease the efficacy of pharmacological therapy.^34^ In addition, analysis of data from the CareRA trial showed a negative correlation between psychosocial burden, as measured by the Short Form 36, Revised Illness Perception Questionnaire and Utrecht Coping List, in that patients with a low psychosocial burden spent longer in remission than patients with a high psychosocial burden (HR = 0.51).^35^

## Potential selection criteria for treatment following first-line anti-TNF failure

**Figure 1.**
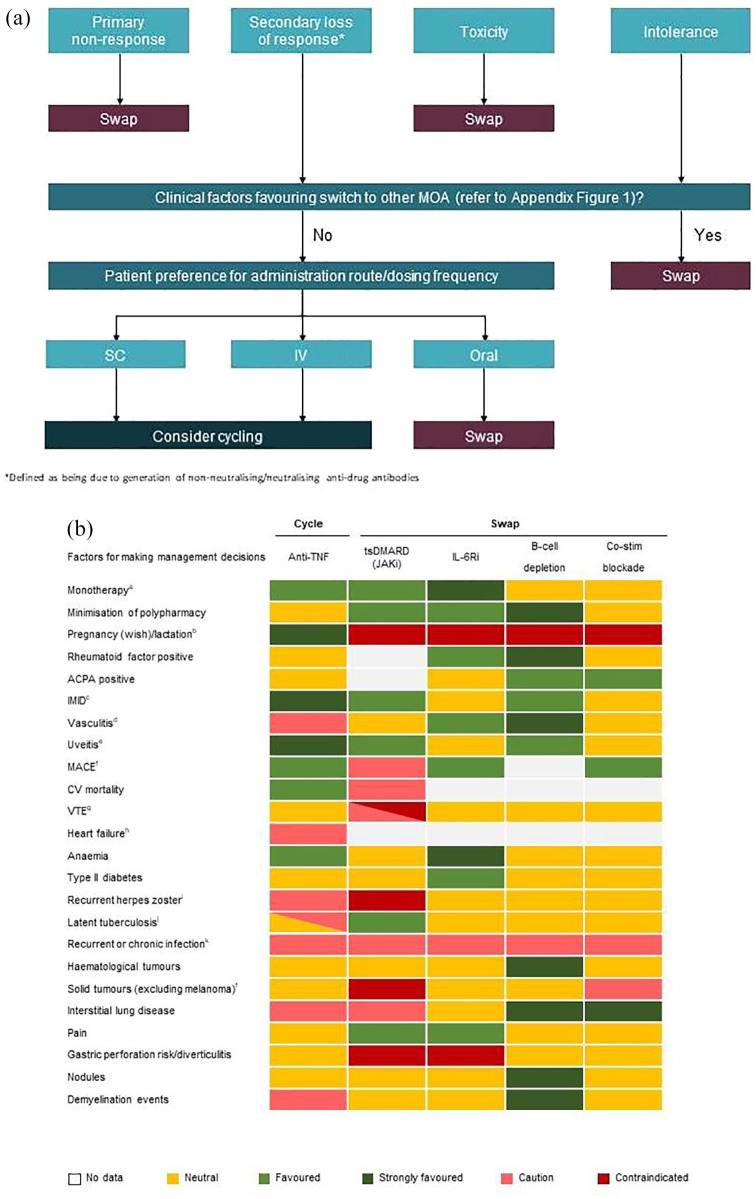
(a) Decision tree for treatment selection following first-line anti-TNF therapy. (b) Treatment options following first-line anti-TNF therapy considering patient characteristics^a^ (supporting references can be found in Supplemental Appendix Figure 1). ACPA, anti-citrullinated peptide antibodies; CV, cardiovascular; IMID, immune-mediated inflammatory diseases; MACE, major adverse cardiovascular event; VTE, venous thromboembolism. ^a^Favoured option if to be used as monotherapy. EULAR guidelines favour IL-6 inhibitors or JAKi as monotherapy in patients who have an intolerance to csDMARDs. ^b^Preferred order of anti-TNFs is certolizumab pegol > etanercept > adalimumab > golimumab > infliximab (based on authors’ experience). The short half-life of JAKi means that a short time needs to elapse before contraception is discontinued, but does not imply that these agents are recommended during either pregnancy or lactation. ^c^TNFi indicated for use in inflammatory bowel disease, ankylosing spondylitis, psoriasis and psoriatic arthritis. Rituximab indicated for use in rheumatoid arthritis, polyangiitis and pemphigus vulgaris. ^d^Anti-TNFs not recommended if vasculitis associated with anti-TNF use, some reports of vascular complications or drug-induced lupus. IL-6Ri recommended if vasculitis is giant cell arteritis. ^e^Monoclonal antibody anti-TNFs to treat uveitis. ^f^Contraindication for JAKi based on ORAL surveillance data for tofacitinib as compared with anti -TNF. The ORAL Surveillance study (NCT02092467) showed that in in highly selected patients with RA ⩾ 50 years of age and ⩾ 1 baseline CV risk factor, there was a numerical difference favouring anti-TNFs compared with tofacitinib in the incidence rates of MACE, VTE and malignancies. However, it is not known if this is a class effect of JAKi, and long-term trials and real-world evidence for tofacitinib have not produced similar signals. Treatment choice should be made through shared decision-making but at the moment patients with risk factors should not be given JAKi if at all possible. ^g^Higher risk of VTE with JAKi if patient has risk factors for VTE, but risk may not be consistent across the class. ^h^Anti-TNFs not recommended if heart failure occurred while on treatment. ^i^Anti-TNFs increase the incidence of herpes zoster but usually not of clinical interest JAKi trials show an increased incidence although this seems less marked with filgotinib. ^j^Infliximab is contraindicated for patients with latent tuberculosis. Routine clinical practice is to screen all patients for latent TB and provide prophylactic treatment. ^k^All bDMARDs and JAKi provide some risk of increased infection. IL-6 inhibitors have a potentially slightly higher risk than other treatments. It should be noted that some therapies offer the possibility of using them at reduced dosages, such as etanercept (25 mg/week) and baricitinib (2 mg/day). The potential for dose reduction should be considered as part of the treatment strategy.

Selection of a treatment for individual patients with RA is influenced by a number of factors. Figure 1(a) provides a decision tree for treatment selection following first-line anti-TNF therapy. This is based upon the heatmap shown in Figure 1(b), which provides the consolidated opinion of the authors on how individual patient and initial therapy characteristics may affect selection of the next treatment option. While there is still an unmet need for robust clinical evidence which supports the most appropriate time point for decision-making regarding change of therapy,^1^ given the debilitating nature of RA, rapid attainment of the selected target endpoint remains critically important to minimise patient disability. In particular, the presence of established comorbidities and/or extra-articular manifestations appear to considerably influence treatment choice^1,36^ with extra-articular inflammatory manifestations of RA occurring in up to 40% of patients.^37^

### Lifestyle and patient preferences

Lifestyle preferences can have a large impact on treatment choice for patients. These can include decisions, such as oral *versus* parenteral administration and the need for refrigeration of certain drugs for frequent travellers and the patient who favours infrequent intravenous (IV) administration.^38^ In addition, the devices that are used to deliver subcutaneous (SC) formulations (prefilled syringes, autoinjectors) can differ between drugs and the patient’s comfort level with a particular device can feed into the decision-making process.^39^ Patient preferences have been previously described for the second-line b/tsDMARD treatment of RA, with treatment effectiveness, route of administration and the probability of severe side effects cited as the most important attributes.^40^ Of note, the possibility of treatment-related psychological side effects, such as anxiety, mood changes, depression or sleep disturbance remain an important factor when selecting treatment, particularly given that more than one-third report previous experience of such events. Taylor *et al.*^40^ reported a preference for oral treatment in older patients, those with RA for < 2 years, and multiple comorbidities. The individual circumstances of patients with RA, including lifestyle (active *vs* sedentary), occupation (e.g. shift work) and living environment may also have an influence on the most suitable treatment choice in terms of route and frequency of administration, design of the device for delivery of SC injections and whether or not refrigeration is required for biologic storage. Given that treatment adherence can impact effectiveness in all disciplines, including RA, the route of administration should be considered when choosing a therapy for patients for whom adherence could be challenging, for example, an IV or SC bDMARD administered at hospital for patients who are older or deemed to be more forgetful, thereby optimising adherence.

### Pregnancy and lactation

For patients who wish to become pregnant, the authors’ clinical experience suggests that the preferred order of treatment selection is certolizumab pegol > etanercept > adalimumab > golimumab > infliximab. Low transfer to breast milk has been shown for infliximab, adalimumab, etanercept and certolizumab. For patients with RA who wish to become pregnant, there is growing evidence on the safety of anti-rheumatic medications in pregnancy and breastfeeding.^41^ Among biologics, anti-TNFs have been most extensively studied and appear reasonably safe with first and second trimester use.^42^ As there is limited evidence for the safe use of rituximab, tocilizumab and abatacept in pregnancy, and JAKi are contraindicated in pregnancy, these agents should be replaced with a more suitable agent prior to conception. Continuation of anti-TNFs should be considered suitable when breastfeeding.

### Age

Age can impact treatment choice in RA through changes in physiological systems and processes, the need for polypharmacy and the increased prevalence of comorbidities.^43^ In addition, dose reductions of the selected therapy may be needed in elderly patients or those with renal impairment, while there are warnings against using tofacitinib in patients aged > 65 years. In older patients, treatment for RA needs to be tailored to factor in these additional concerns.

### Comorbidities

The EULAR guidelines state that ‘Treatment decisions are based on disease activity, safety issues and other patient factors, such as comorbidities and progression of structural damage’.^1^

The use of anti-TNFs in patients with RA has been associated with a significant reduction in cardiovascular (CV) disease risk, along with decreased risk of myocardial infarction compared those receiving csDMARD therapy over the medium term, which might be attributed to a direct action of TNF inhibition on the atherosclerotic process or simply better overall disease control.^44–46^ A head-to-head study showed no increase in rates of major adverse cardiac events (MACEs) using tocilizumab *versus* anti-TNFs (etanercept).^47^

Events of venous thromboembolism (VTE) have been documented in clinical trials of JAK inhibitors in patients with RA.^8–11^ Of note, VTEs, including pulmonary embolism, are more commonly reported in patients with a high risk for these events, such as those with a previous history of thromboembolic events, those with high body mass index, those with hormone replacement therapy and higher age.^8–11,48^

Obesity is a risk and severity factor in rheumatic diseases.^49^ Clinical experience suggests that the presence of obesity is generally only of practical relevance for those few treatments, such as golimumab, where a higher dose option is possible. However, a recent analysis of 10,593 patients contained within the German RABBIT registry showed that obesity (> 30 kg/m^2^) has a negative effect on the effectiveness of cytokine-targeted (anti-TNFs and tocilizumab) but not cell-targeted (rituximab and abatacept) therapies in daily practice, and that these effects were more noticeable in women compared with men.^50^

### Smoking

Smoking/tobacco use can also affect response to treatment in patients with RA.^51^ Several studies have shown that the response to anti-TNF therapies is reduced in smokers and that they are less likely to achieve disease targets. Smoking has also been shown to reduce the impact of methotrexate but not of rituximab.^52,53^ There seems to be minimal if any data published for IL-6 inhibitors, abatacept and JAKi.

### Pulmonary disease

RA-associated pulmonary complications are common and cause 10–20% of overall mortality.^54^ Interstitial lung disease (ILD) is a pulmonary manifestation that may be related to the RA inflammatory process itself, infectious complications or the selected treatment regimen.^55^ Thus, treatments with minimal impact on pulmonary function may need to be selected in some patients. Published evidence suggests a potential role for anti-TNF agents in causing or worsening ILD in RA patients, while the use of rituximab may improve the condition.^55,56^ Data for the use of JAK inhibitors or IL-6 receptor antagonists are scarce. Recent studies have suggested that abatacept may be beneficial in RA patients with ILD. In 236 patients with RA and ILD treated with abatacept, Fernández-Díaz *et al.*^57^ showed that after a follow-up of 12 months, dyspnoea, forced vital capacity, diffusion lung capacity for carbon monoxide and high-resolution computed tomography (CT) were stable in 75–90% of patients, and corticosteroid use was reduced. A systematic review concluded that abatacept led to significantly lower ILD worsening rates compared with anti-TNFs with a 90% reduction in relative risk of deterioration of ILD at 24 months of follow-up.^58^

### Pain

Emerging evidence suggests that where pain is a predominant symptom in RA, there may be some advantages in swapping to a JAKi over cycling anti-TNFs^59^ as JAKi appear to have beneficial effects on inflammatory and non-inflammatory pain in general.^60^ Such findings are consistent with the overarching involvement of the JAK-signal transducer and activator of transcription pathway in mediating the action, expression, and regulation of multiple pro- and anti-inflammatory cytokines.^61^ It is also possible that the use of an oral medication could reduce the perception of pain in patients who are needle phobic. Further research is required to elucidate the precise mechanisms by which JAKi reduce pain generation. Another complicating factor with respect to pain management in RA patients is the presence of fibromyalgia, which occurs in a significant proportion of patients.^62^ If pain is still an issue once the systemic and local inflammatory component of RA has been addressed through the use of targeted therapy, then other alternative wellness strategies are required.^63^ The clinician needs to be careful that they avoid potential overtreatment of the patient with immunosuppressive therapy, narrowing the benefit: risk ratio and increasing the risk of adverse events, such as infections.

### CV and cancer risk factors

There is an increased risk of CV disease in patients with RA,^24^ where systemic inflammation is thought to directly contribute to CV disease risk and subclinical CV disease may be present from the early phase of RA. Studies have shown that the CV morbidity and mortality risk of RA patients has decreased over the past two decades and have suggested that this may be due to better treatment options and closer and earlier control of the disease.^64–66^ Indeed, a treat-to-target approach has been shown to result in reduced levels of CV disease biomarkers and risk factors.^67,68^ However, CV risk does remain in RA patients and, therefore, treatment strategies should incorporate its management through targeting chronic inflammation and traditional CV disease risk factors.^69,70^

Preliminary findings from a prospective, randomised, post-authorisation safety study of tofacitinib (A3921133 / NCT02092467) comparing outcomes between treatments in patients with RA who were aged ⩾ 50 years and had ⩾ 1 additional CV risk factor showed an increased rate of malignancies for tofacitinib (IR/100 PY, 1.13) relative to anti-TNFs (IR/100 PY, 0.77).^71^ However, the reported malignancy rates in the phase III trials for tofacitinib, baricitinib, upadacitinib and filgotinib were within expected boundaries for a rheumatoid population and, therefore, it is unclear at present whether these findings might be more generalisable to the use of the JAKi class as a whole. Reported rates of MACE in the tofacitinib post-authorisation study (IR/100 PY) were 0.98 for tofacitinib compared with 0.73 for anti-TNFs. Post hoc analyses showed that most MACE and malignancies occurred in patients who were aged ⩾ 65 years or had ever smoked. Possible hypotheses to explain these findings include a protective effect of TNFis. However, it should be noted that the higher incidence rate (IR) observed with tofacitinib in this population with baseline risk factors remains within the wide boundaries (IR = 0.2–2.4) reported for MACE in epidemiological studies within the general RA population. Disclosure and analysis of the full data set may shed more light on the relationship between baseline characteristics and the occurrence of adverse events.

## Biomarkers

The current EULAR guidelines state that ‘The major weakness of our current treatment approaches is the lack of biomarkers for immediate stratification of an individual patient to the most appropriate drug’.^1^ While such considerations highlight the ongoing need for predictive biomarkers, the guidelines note that the presence of rheumatoid factor and/or anti-citrullinated peptide antibodies (ACPAs), particularly at high levels, remain useful prognostic factors in patients with RA. As ACPA positivity is associated with a severe erosive phenotype and higher mortality rate compared with seronegative RA, this may influence treatment choice given the favourable response to biologics targeting pathways involving autoantibody producing cells.^72^ While ACPA positivity has an inconsistent relationship with the effectiveness of anti-TNFs, it has been consistently associated with response in rituximab-treated patients with RA.^73^ For B-cell depleting bDMARDs, and a lesser extent abatacept, response is better in seropositive patients.^74,75^ Response to abatacept was better than that to adalimumab in patients with a shorter disease course.^76^ Apart from these data, biomarkers currently have little place in informing management decisions. Several investigators are attempting to use machine learning techniques to determine which currently measured clinical factors can help identify those patients most likely to respond to a given therapy. One study, using data taken from 1204 patients treated with bDMARDs from the Korean College of Rheumatology Biologics and Targeted Therapy Registry, showed that different patient characteristics were predictive of remission for different therapies, including age for adalimumab, rheumatoid factor for etanercept and C-reactive protein (CRP) for tocilizumab.^77^ Another study used machine learning to identify blood biomarkers that could be used to predict responses to sarilumab with data being taken from the MOBILITY, MONARCH, TARGET and ASCERTAIN clinical trials.^78^ The presence of ACPA in combination with CRP levels > 12.3 mg/L predicted response to sarilumab for many efficacy parameters including ACR20, ACR70 and DAS28-CRP. Interestingly, the rule was not as effective for predicting response for patients from TARGET that recruited patients refractory to TNFis.

### Therapeutic drug monitoring

Therapeutic decision-making within RA does not include routine monitoring of ADAs or drug concentrations in patient serum.^1^ However, therapeutic drug monitoring may be considered to aid cycling or swapping decisions.^79^ For those patients who report loss of response, therapeutic drug monitoring may help to identify those patients who are more likely to benefit from cycling within class.^80^ High drug levels and absence of ADA would suggest that switching to an alternative MoA would be advisable, whereas low drug levels and high ADA would suggest switching within class.

### Cost-effectiveness

The 2019 EULAR guidelines recognise cost as a factor that physicians need to consider when choosing a suitable treatment for their patients.^1^ Manders *et al.*^81^ assessed the cost-effectiveness of treatments with differing modes of action following failure of anti-TNFs in a randomised trial and reported rituximab to be a favourable option based on clinical effectiveness and associated cost over a 12-month period. Studies have assessed the cost-effectiveness of DMARD treatment sequences in patients with RA from the perspectives of a US healthcare database or the Finnish health system with varying conclusions.^82,83^ Anti-TNFs were reported to have the lowest costs and highest quality-adjusted life-year (QALYs) (*vs* other biologics), and were deemed to be the most cost-effective treatment option in RA.^83^ Using a US-based administrative-claims database, Karpes Matusevich *et al.*^19^ reported that swapping to non-anti-TNF targeted agents was cost-effective at the US$100,000 per QALY threshold following failure of anti-TNF treatment in patients with RA, although differences in the design, key assumptions, and model structure chosen had a major impact on the individual study conclusions. This study highlighted the need for further studies to evaluate cost-effectiveness with switching choices other than rituximab or IV abatacept, to better reflect current clinical practices, of longer-term studies on the progression of RA, of RA costs over time and for greater standardisation and transparency in the reporting of economic evaluation studies.

### Biosimilars

The availability of biosimilars, mainly anti-TNFs, for use in the treatment of RA appears to be further driving cost reductions. Müskens *et al.*^84^ reported the average cost per patient treated with biologics to decrease following the introduction of biosimilars, with a persistent trend being seen. Based on the estimated budget impact on rheumatology specialities in the UK hospitals, Aladul *et al.*^85^ reported that when a biosimilar is available for a directly comparable branded molecule, price is the key influencing factor in the prescribing of a specific product. In addition, the use of biosimilars may lead to improved access to biologics as illustrated by the recent modification of recommendations within the United Kingdom to extend coverage of TNFis to moderate RA patients following the introduction of more affordable biosimilars.^86^ The use of anti-TNF biosimilars that move to SC *versus* IV modes of administration may provide a novel opportunity to support treatment adherence given that they appear to offer similar efficacy without any change in safety signals, while also offering high usability and patient convenience, along with a potential cost saving.^87^

### COVID-19

The ongoing COVID-19 pandemic has the potential to influence treatment choice in patients with RA. However, RA patients with well-controlled disease are at less risk of morbidity or mortality from severe acute respiratory syndrome coronavirus 2 (SARS-CoV-2) infection than those who have highly active disease. Furthermore, patients receiving targeted therapies for RA do not appear to be at a greatly increased risk of acquiring coronavirus disease 2019 (COVID-19).^88^ Thus, patients with RA should receive treatment as usual with a view to optimum disease control. However, recent evidence from a large observational cohort suggests that patients treated with rituximab may have a higher risk for developing severe COVID-19 than those given other treatments.^89^ As with other infections, a temporary cessation of targeted therapy may be advised in the event of contracting SARS-CoV-2.

## Conclusion

RA is a lifelong, potentially debilitating condition necessitating a tailored and cost-effective approach to its management. As many patients will experience an IR to a given therapy at some point during their disease, it is essential to adjust pharmacotherapy as required so that the best outcomes for the patient are achieved within the shortest possible timeframe. It is hoped that the information contained within the current review will assist clinicians in selecting the most appropriate therapy for their patients following anti-TNF-IR. Indeed, when taking the factors into account that are relevant for an individual patient, cycling to another anti-TNF may be an effective option in many situations with the additional benefit of cost-effectiveness compared with some other strategies.

## Supplemental Material

sj-docx-2-tab-10.1177_1759720X221114101 – Supplemental material for Managing inadequate response to initial anti-TNF therapy in rheumatoid arthritis: optimising treatment outcomes Click here for additional data file. Supplemental material, sj-docx-2-tab-10.1177_1759720X221114101 for Managing inadequate response to initial anti-TNF therapy in rheumatoid arthritis: optimising treatment outcomes by Peter C. Taylor, Marco Matucci Cerinic, Rieke Alten, Jérôme Avouac and Rene Westhovens in Therapeutic Advances in Musculoskeletal Disease

sj-docx-3-tab-10.1177_1759720X221114101 – Supplemental material for Managing inadequate response to initial anti-TNF therapy in rheumatoid arthritis: optimising treatment outcomes Click here for additional data file. Supplemental material, sj-docx-3-tab-10.1177_1759720X221114101 for Managing inadequate response to initial anti-TNF therapy in rheumatoid arthritis: optimising treatment outcomes by Peter C. Taylor, Marco Matucci Cerinic, Rieke Alten, Jérôme Avouac and Rene Westhovens in Therapeutic Advances in Musculoskeletal Disease

sj-pptx-1-tab-10.1177_1759720X221114101 – Supplemental material for Managing inadequate response to initial anti-TNF therapy in rheumatoid arthritis: optimising treatment outcomes Click here for additional data file. Supplemental material, sj-pptx-1-tab-10.1177_1759720X221114101 for Managing inadequate response to initial anti-TNF therapy in rheumatoid arthritis: optimising treatment outcomes by Peter C. Taylor, Marco Matucci Cerinic, Rieke Alten, Jérôme Avouac and Rene Westhovens in Therapeutic Advances in Musculoskeletal Disease

## Declarations

**Ethics approval and consent to participate:** Not applicable.

**Consent for publication:** Not applicable.

**Availability of data and materials:** Not applicable.
