# Supplementary material for: Managing inadequate response to initial anti-TNF therapy in rheumatoid arthritis: optimising treatment outcomes
Source: Ther Adv Musculoskelet Dis. 2022 Aug 16;14:1759720X221114101. doi: 10.1177/1759720X221114101 (PMC9386864; doi:10.1177/1759720X221114101)
Supplement: sj-docx-2-tab-10.1177_1759720X221114101 – Supplemental material for Managing inadequate response to initial anti-TNF therapy in rheumatoid arthritis: optimising treatment outcomes [file sj-docx-2-tab-10.1177_1759720X221114101.docx]

**Appendix Table 1.** Results from studies focusing on primary or secondary non-response

| Reference | Design | N | Definition of response | Results |
| --- | --- | --- | --- | --- |
| *Primary non-response* |  |  |  |  |
| Chatzidionysiou et al. 2015^1^ | Registry:  anti-TNF→anti-TNF | 952 | LDA (≤3.2 DAS28) at 6 months post swap/cycling | 29% of patients had good clinical responses (28-joint Disease Activity Score [DAS28] remission or low disease activity [LDA]) |
| Bessette et al. 2017^2^ | Single arm, interventional study: adalimumab→etanercept | 86 | Proportion of patients achieving ACR20 | 20% of patients achieved an ACR20 response at Wk 24 post cycling |
| Smolen et al. 2016^3^ | Randomised, double/single blind: adalimumab→certolizumab certolizumab→adalimumab | 915 | LDA (≤3.2 DAS28 (ESR)) or DAS28(ESR) ≥1.2 at 12 weeks post cycling | Swapping from CZP to ADL, or ADL to CZP resulted in ~60% of patients achieving LDA (≤3.2 DAS28 (ESR)) or DAS28(ESR) ≥1.2 |
| Gottenberg et al. 2016^4^ | Multicentre, open, parallel-group, randomised:  anti-TNF→anti-TNF or non-anti-TNF | 300 | Good (decrease in DAS28-ESR of> 1.2 points, resulting in a score of ≤3.2) or moderate (decrease of >0.6, and resulting in a score of ≤5.1) EULAR response | 69% of patients achieved an effective clinical response with a non-TNF biologic vs 52% of patients who received a second anti-TNF drug |
| *Secondary non-response* |  |  |  |  |
| Hyrich et al. 2008^5^ | Registry: anti-TNF→anti-TNF | 868 | Change in HAQ score over 12 months | 36% of patients who cycled to another anti-TNF demonstrated a ≥0.22 U improvement HAQ score by month 12 |
| Chatzidionysiou et al. 2015^1^ | Registry: anti-TNF→anti-TNF | 952 | LDA (≤3.2 DAS28) at 6 months post swap/cycling | 40% of patients had good clinical responses (28-joint Disease Activity Score [DAS28] remission or low disease activity [LDA]) |
| Navarro-Sarabia et al. 2009^6^ | Observational, cohort: anti-TNF→anti-TNF | 417 | Not provided | Patients swapping to a second anti-TNF experienced a 1.1-point decrease in DAS28 score (P<0.0001) and a 0.21 U decrease in HAQ (P<0.004) compared with baseline at cycling |
| Bombardieri et al. 2007^7^ | Open-label: Etanercept/infliximab→adalimumab | 899 | LDA (<3.2 DAS28) at 12 weeks, EULAR response | At Wk 12 60% of patients had an ACR20, 33% had an ACR50 and 76% had a moderate EULAR response |
| Bessette et al. 2017^2^ | Single arm, interventional: adalimumab→etanercept | 86 | Proportion of patients achieving ACR20 | 40% of patients achieved an ACR20 response at Wk 24 post cycling |
| Fleischmann et al. 2014^8^ | Single arm, interventional: etanercept/adalimumab→infliximab | 203 | EULAR response at Wk 26 | At Wk 26 36% of patients had an ACR20, 18% an ACR50 and 65% a EULAR response |
| Koike et al. 2012^9^ | Post-marketing surveillance: infliximab→etanercept | 908 | EULAR response | At Wk 24 >80% of patients had a good or moderate EULAR response |
| Weinblatt et al. 2012^10^ | Randomised, double-blind:  anti-TNF→certolizumab | 1063 | Proportion of patients achieving ACR20 at Wk 12 | At Wk 12 47% of patients achieved ACR20 and 22% of patients achieved ACR50 |
| Smolen et al. 2009^11^ | Randomised, double-blind: anti-TNF→golimumab | 461 | Proportion of patients achieving ACR20 at Wk 14 | At Wk 14 37% of patients achieved ACR20 and 18% of patients achieved ACR50, values at Wk 24 were 39% and 19% respectively |
| Favalli et al. 2020^12^ | Retrospective: cycling anti-TNF  etanercept→adalimumab | 117 | Reduction in DAS28, LDA (<3.2) and EULAR response at month 12 | At Month 12 DAS28 had reduced from 4.97 to 3.50, 27% of patients were in remission, 24% had LDA and 52% had a EULAR response |
| Genovese et al. 2005^13^ | Randomised, double-blind, placebo-controlled: anti-TNF→abatacept | 391 | ACR20, HAQ at 6 months | At 6 months following swap, 50% of patients had achieved an ACR20 and 20% ACR50. 47% of patients achieved an improvement of 0.3 U in HAQ |
| Cohen et al. 2006^14^ | Randomised, double-blind, placebo-controlled: anti-TNF→rituximab | 520 | ACR20 at 24 weeks | 51% of patients achieved an ACR20 at 24 weeks and 27% an ACR50 |
| Emery et al. 2008^15^ | Randomised, double-blind, placebo-controlled: anti-TNF→tocilizumab | 499 | ACR20 at 24 weeks | 50% of patients achieved an ACR20 at 24 weeks and 28% an ACR50 |
| Fleischmann et al. 2017^16^ | Randomised, double-blind, placebo-controlled: anti-TNF→sarilumab | 546 | ACR20 at Wk 24 and HAQ DI at Wk 12 | 61% of patients achieved an ACR20 at 24 weeks and a 0.6-point reduction in HAQ DI at 12 weeks |
| Burmester et al. 2013^17^ | Randomised, double-blind: anti-TNF→tofacitinib | 399 | ACR20, HAQ DI and proportion of patients with DAS28-4ESR <2.6 at Month 3 | At Month 3 42% of patients had an ACR20, there was a -0.43-point reduction in HAQ DI and 7% of patients had DAS28-4ESR <2.6 |
| Genovese et al. 2016^18^ | Randomised, double-blind, placebo-controlled: bDMARD→baricitinib | 527 | ACR20, HAQ DI, DAS28-CRP <2.6, SDAI ≤3.3 at Week 12 | At Week 12 55% of patients had an ACR20, a ~0.4-point decrease in HAQ DI and ~1.85-point decrease in DAS28-CRP |
| *Non-response or intolerance* |  |  |  |  |
| Fleischmann et al. 2020^19^ | Randomised, double-blind, placebo/active comparator-controlled:  adalimumab→upadacitinib /  upadacitinib→adalimumab | 1629 | 20% improvement in TJC and SJC (non-responders)  20% improvement in CDAI at Wk 26 (incomplete responders) | CDAI low disease activity achieved by 36% and 47% of non-responders and 45% and 58% of incomplete-responders switched to adalimumab and upadacitinib, respectively, 6 months post switch |
| Humby et al. 2021^20^ | Randomised, open-label, active-comparator controlled:  bDMARD→rituximab or tocilizumab | 164 | 50% improvement in CDAI from baseline | In patients classified as B-cell poor via RNA sequencing tocilizumab was associated with a significantly higher response rate than rituximab (63% vs 36%, p=0.035) |

ACR, American College of Rheumatology; CRP, C-reactive protein; DAS28, Disease Activity Score 28 joints; ESR, erythrocyte sedimentation rate; EULAR, European League Against Rheumatism; HAQ DI, Health Assessment Questionnaire Disability Index; LDA, low disease activity; TNF, tumour necrosis factor; Wk, week.
