# Supplementary material for: Managing inadequate response to initial anti-TNF therapy in rheumatoid arthritis: optimising treatment outcomes
Source: Ther Adv Musculoskelet Dis. 2022 Aug 16;14:1759720X221114101. doi: 10.1177/1759720X221114101 (PMC9386864; doi:10.1177/1759720X221114101)
Supplement: sj-docx-3-tab-10.1177_1759720X221114101 – Supplemental material for Managing inadequate response to initial anti-TNF therapy in rheumatoid arthritis: optimising treatment outcomes [file sj-docx-3-tab-10.1177_1759720X221114101.docx]

**Appendix**

**Managing inadequate response to initial anti-TNF therapy in rheumatoid arthritis: optimising treatment outcomes**

**Peter C. Taylor,^1^ Marco Matucci Cerinic,^2,3^ Rieke Alten,^4^ Jérôme Avouac,^5^ Rene Westhovens^6^**

1. *Botnar Research Centre, Nuffield Department of Orthopaedics, Rheumatology and Musculoskeletal Sciences, University of Oxford, UK*
2. *Department of Experimental and Clinical Medicine, University of Florence, Florence, Italy,*
3. *Unit of Immunology, Rheumatology, Allergy and Rare Diseases (UnIRAR), IRCCS San Raffaele Hospital, Milan, Italy*
4. *Department of Internal Medicine, Rheumatology, Clinical Immunology and Osteology, Schlosspark-Klinik University Medicine Berlin, Berlin, Germany*
5. *AP-HP.Centre, Université de Paris, Hôpital Cochin, Service de Rhumatologie, Paris, France*
6. *Department of Development and Regeneration, Skeletal Biology and Engineering Research Center, Division of Rheumatology, KU Leuven, Leuven, Belgium*

**Correspondence to:** Professor Peter C. Taylor, Botnar Research Centre, Nuffield Department of Orthopaedics, Rheumatology and Musculoskeletal Sciences, University of Oxford, Old Rd, Headington, Oxford OX3 7LD, UK

**E-mail:** peter.taylor@kennedy.ox.ac.uk

**Appendix Table 1.** Results from studies focusing on primary or secondary non-response

| Reference | Design | N | Definition of response | Results |
| --- | --- | --- | --- | --- |
| *Primary non-response* |  |  |  |  |
| Chatzidionysiou et al. 2015^1^ | Registry:  anti-TNF→anti-TNF | 952 | LDA (≤3.2 DAS28) at 6 months post swap/cycling | 29% of patients had good clinical responses (28-joint Disease Activity Score [DAS28] remission or low disease activity [LDA]) |
| Bessette et al. 2017^2^ | Single arm, interventional study: adalimumab→etanercept | 86 | Proportion of patients achieving ACR20 | 20% of patients achieved an ACR20 response at Wk 24 post cycling |
| Smolen et al. 2016^3^ | Randomised, double/single blind: adalimumab→certolizumab certolizumab→adalimumab | 915 | LDA (≤3.2 DAS28 (ESR)) or DAS28(ESR) ≥1.2 at 12 weeks post cycling | Swapping from CZP to ADL, or ADL to CZP resulted in ~60% of patients achieving LDA (≤3.2 DAS28 (ESR)) or DAS28(ESR) ≥1.2 |
| Gottenberg et al. 2016^4^ | Multicentre, open, parallel-group, randomised:  anti-TNF→anti-TNF or non-anti-TNF | 300 | Good (decrease in DAS28-ESR of> 1.2 points, resulting in a score of ≤3.2) or moderate (decrease of >0.6, and resulting in a score of ≤5.1) EULAR response | 69% of patients achieved an effective clinical response with a non-TNF biologic vs 52% of patients who received a second anti-TNF drug |
| *Secondary non-response* |  |  |  |  |
| Hyrich et al. 2008^5^ | Registry: anti-TNF→anti-TNF | 868 | Change in HAQ score over 12 months | 36% of patients who cycled to another anti-TNF demonstrated a ≥0.22 U improvement HAQ score by month 12 |
| Chatzidionysiou et al. 2015^1^ | Registry: anti-TNF→anti-TNF | 952 | LDA (≤3.2 DAS28) at 6 months post swap/cycling | 40% of patients had good clinical responses (28-joint Disease Activity Score [DAS28] remission or low disease activity [LDA]) |
| Navarro-Sarabia et al. 2009^6^ | Observational, cohort: anti-TNF→anti-TNF | 417 | Not provided | Patients swapping to a second anti-TNF experienced a 1.1-point decrease in DAS28 score (P<0.0001) and a 0.21 U decrease in HAQ (P<0.004) compared with baseline at cycling |
| Bombardieri et al. 2007^7^ | Open-label: Etanercept/infliximab→adalimumab | 899 | LDA (<3.2 DAS28) at 12 weeks, EULAR response | At Wk 12 60% of patients had an ACR20, 33% had an ACR50 and 76% had a moderate EULAR response |
| Bessette et al. 2017^2^ | Single arm, interventional: adalimumab→etanercept | 86 | Proportion of patients achieving ACR20 | 40% of patients achieved an ACR20 response at Wk 24 post cycling |
| Fleischmann et al. 2014^8^ | Single arm, interventional: etanercept/adalimumab→infliximab | 203 | EULAR response at Wk 26 | At Wk 26 36% of patients had an ACR20, 18% an ACR50 and 65% a EULAR response |
| Koike et al. 2012^9^ | Post-marketing surveillance: infliximab→etanercept | 908 | EULAR response | At Wk 24 >80% of patients had a good or moderate EULAR response |
| Weinblatt et al. 2012^10^ | Randomised, double-blind:  anti-TNF→certolizumab | 1063 | Proportion of patients achieving ACR20 at Wk 12 | At Wk 12 47% of patients achieved ACR20 and 22% of patients achieved ACR50 |
| Smolen et al. 2009^11^ | Randomised, double-blind: anti-TNF→golimumab | 461 | Proportion of patients achieving ACR20 at Wk 14 | At Wk 14 37% of patients achieved ACR20 and 18% of patients achieved ACR50, values at Wk 24 were 39% and 19% respectively |
| Favalli et al. 2020^12^ | Retrospective: cycling anti-TNF  etanercept→adalimumab | 117 | Reduction in DAS28, LDA (<3.2) and EULAR response at month 12 | At Month 12 DAS28 had reduced from 4.97 to 3.50, 27% of patients were in remission, 24% had LDA and 52% had a EULAR response |
| Genovese et al. 2005^13^ | Randomised, double-blind, placebo-controlled: anti-TNF→abatacept | 391 | ACR20, HAQ at 6 months | At 6 months following swap, 50% of patients had achieved an ACR20 and 20% ACR50. 47% of patients achieved an improvement of 0.3 U in HAQ |
| Cohen et al. 2006^14^ | Randomised, double-blind, placebo-controlled: anti-TNF→rituximab | 520 | ACR20 at 24 weeks | 51% of patients achieved an ACR20 at 24 weeks and 27% an ACR50 |
| Emery et al. 2008^15^ | Randomised, double-blind, placebo-controlled: anti-TNF→tocilizumab | 499 | ACR20 at 24 weeks | 50% of patients achieved an ACR20 at 24 weeks and 28% an ACR50 |
| Fleischmann et al. 2017^16^ | Randomised, double-blind, placebo-controlled: anti-TNF→sarilumab | 546 | ACR20 at Wk 24 and HAQ DI at Wk 12 | 61% of patients achieved an ACR20 at 24 weeks and a 0.6-point reduction in HAQ DI at 12 weeks |
| Burmester et al. 2013^17^ | Randomised, double-blind: anti-TNF→tofacitinib | 399 | ACR20, HAQ DI and proportion of patients with DAS28-4ESR <2.6 at Month 3 | At Month 3 42% of patients had an ACR20, there was a -0.43-point reduction in HAQ DI and 7% of patients had DAS28-4ESR <2.6 |
| Genovese et al. 2016^18^ | Randomised, double-blind, placebo-controlled: bDMARD→baricitinib | 527 | ACR20, HAQ DI, DAS28-CRP <2.6, SDAI ≤3.3 at Week 12 | At Week 12 55% of patients had an ACR20, a ~0.4-point decrease in HAQ DI and ~1.85-point decrease in DAS28-CRP |
| *Non-response or intolerance* |  |  |  |  |
| Fleischmann et al. 2020^19^ | Randomised, double-blind, placebo/active comparator-controlled:  adalimumab→upadacitinib /  upadacitinib→adalimumab | 1629 | 20% improvement in TJC and SJC (non-responders)  20% improvement in CDAI at Wk 26 (incomplete responders) | CDAI low disease activity achieved by 36% and 47% of non-responders and 45% and 58% of incomplete-responders switched to adalimumab and upadacitinib, respectively, 6 months post switch |
| Humby et al. 2021^20^ | Randomised, open-label, active-comparator controlled:  bDMARD→rituximab or tocilizumab | 164 | 50% improvement in CDAI from baseline | In patients classified as B-cell poor via RNA sequencing tocilizumab was associated with a significantly higher response rate than rituximab (63% vs 36%, p=0.035) |

ACR, American College of Rheumatology; CRP, C-reactive protein; DAS28, Disease Activity Score 28 joints; ESR, erythrocyte sedimentation rate; EULAR, European League Against Rheumatism; HAQ DI, Health Assessment Questionnaire Disability Index; LDA, low disease activity; TNF, tumour necrosis factor; Wk, week.

**References: Appendix Table 1**

1. Chatzidionysiou K, Askling J, Eriksson J, et al. Effectiveness of TNF inhibitor switch in RA: results from the national Swedish register. *Ann Rheum Dis* 2015;74:890–6.
2. Bessette L, Khraishi M, Kivitz AJ, et al. Single-arm study of etanercept in adult patients with moderate to severe rheumatoid arthritis who failed adalimumab treatment. *Rheumatol Ther* 2017;4:391–404.
3. Smolen JS, Burmester GR, Combe B, al. Head-to-head comparison of certolizumab pegol versus adalimumab in rheumatoid arthritis: 2-year efficacy and safety results from the randomised EXXELARATE study. *Lancet* 2016;388:2763–74.
4. Hyrich KL, Lunt M, Dixon WG, et al. Effects of switching between anti-TNF therapies on HAQ response in patients who do not respond to their first anti-TNF drug. *Rheumatology (Oxford)* 2008;47:1000–5.
5. Navarro-Sarabia F, Ruiz-Montesinos D, Hernandez B, et al. DAS-28-based EULAR response and HAQ improvement in rheumatoid arthritis patients switching between TNF antagonists. *BMC Musculoskelet Disord* 2009;10:9.
6. Gottenberg JE, Brocq O, Perdriger A, et al. Non-TNF-targeted biologic vs a second anti-TNF drug to treat rheumatoid arthritis in patients with insufficient response to a first anti-TNF drug: A randomized clinical trial. *JAMA* 2016;316:1172–80.
7. Bombardieri S, Ruiz AA, Fardellone P, et al. Effectiveness of adalimumab for rheumatoid arthritis in patients with a history of TNF-antagonist therapy in clinical practice. *Rheumatology (Oxford)* 2007;46:1191–9.
8. Fleischmann R, Goldman JA, Leirisalo-Repo M, et al. Infliximab efficacy in rheumatoid arthritis after an inadequate response to etanercept or adalimumab: results of a target-driven active switch study. *Curr Med Res Opin* 2014;30:2139–49.
9. Koike T, Harigai M, Inokuma S, et al. Safety and effectiveness of switching from infliximab to etanercept in patients with rheumatoid arthritis: results from a large Japanese postmarketing surveillance study. *Rheumatol Int* 2012;32:1617–24.
10. Weinblatt ME, Fleischmann R, Huizinga TW, et al. Efficacy and safety of certolizumab pegol in a broad population of patients with active rheumatoid arthritis: results from the REALISTIC phase IIIb study. *Rheumatology (Oxford)* 2012;51:2204–14.
11. Smolen JS, Kay J, Doyle MK, et al. Golimumab in patients with active rheumatoid arthritis after treatment with tumour necrosis factor alpha inhibitors (GO-AFTER study): a multicentre, randomised, double-blind, placebo-controlled, phase III trial. *Lancet* 2009;374:210–21.
12. Favalli EG, Becciolini A, Carletto A, et al. Efficacy and retention rate of adalimumab in rheumatoid arthritis and psoriatic arthritis patients after first-line etanercept failure: the FEARLESS cohort. *Rheumatol Int* 2020;40:263–72.
13. Genovese MC, Becker JC, Schiff M, et al. Abatacept for rheumatoid arthritis refractory to tumor necrosis factor alpha inhibition. *N Engl J Med* 2005;353:1114–23.
14. Cohen SB, Emery P, Greenwald MW, et al. Rituximab for rheumatoid arthritis refractory to anti-tumor necrosis factor therapy: Results of a multicenter, randomized, double-blind, placebo-controlled, phase III trial evaluating primary efficacy and safety at twenty-four weeks. *Arthritis Rheum* 2006;54:2793–806.
15. Emery P, Keystone E, Tony HP, et al. IL-6 receptor inhibition with tocilizumab improves treatment outcomes in patients with rheumatoid arthritis refractory to anti-tumour necrosis factor biologicals: results from a 24-week multicentre randomised placebo-controlled trial. *Ann Rheum Dis* 2008;67:1516–23.
16. Fleischmann R, van Adelsberg J, Lin Y, et al. Sarilumab and nonbiologic disease-modifying antirheumatic drugs in patients with active rheumatoid arthritis and inadequate response or intolerance to tumor necrosis factor inhibitors. *Arthritis Rheumatol* 2017;69:277–90.
17. Burmester GR, Blanco R, Charles-Schoeman C, et al. Tofacitinib (CP-690,550) in combination with methotrexate in patients with active rheumatoid arthritis with an inadequate response to tumour necrosis factor inhibitors: a randomised phase 3 trial. *Lancet* 2013;381:451–60.
18. Genovese MC, Kremer J, Zamani O, et al. Baricitinib in patients with refractory rheumatoid arthritis. *N Engl J Med* 2016;374:1243–52.

**Appendix Figure 1.** **Treatment options following first line anti-TNF therapy considering patient characteristics^a^**

**
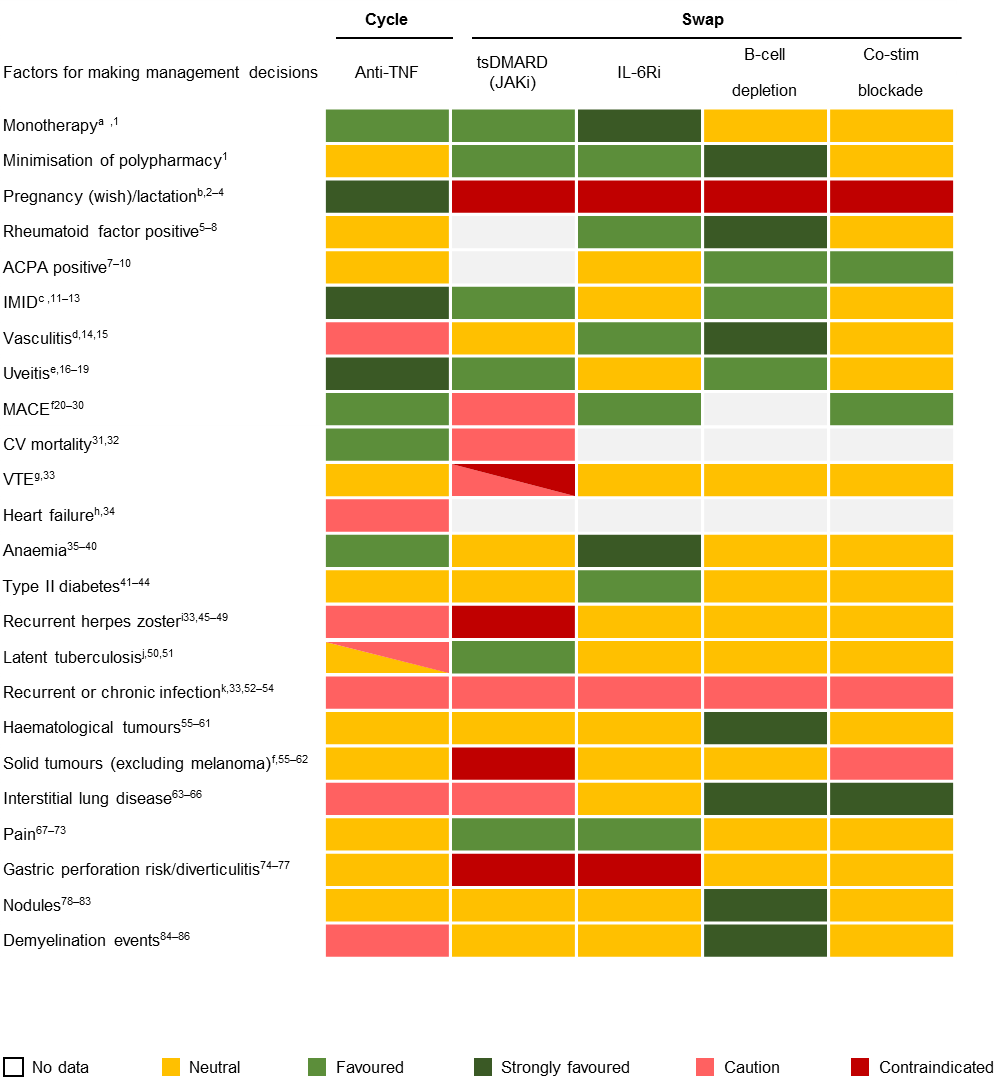
**

**
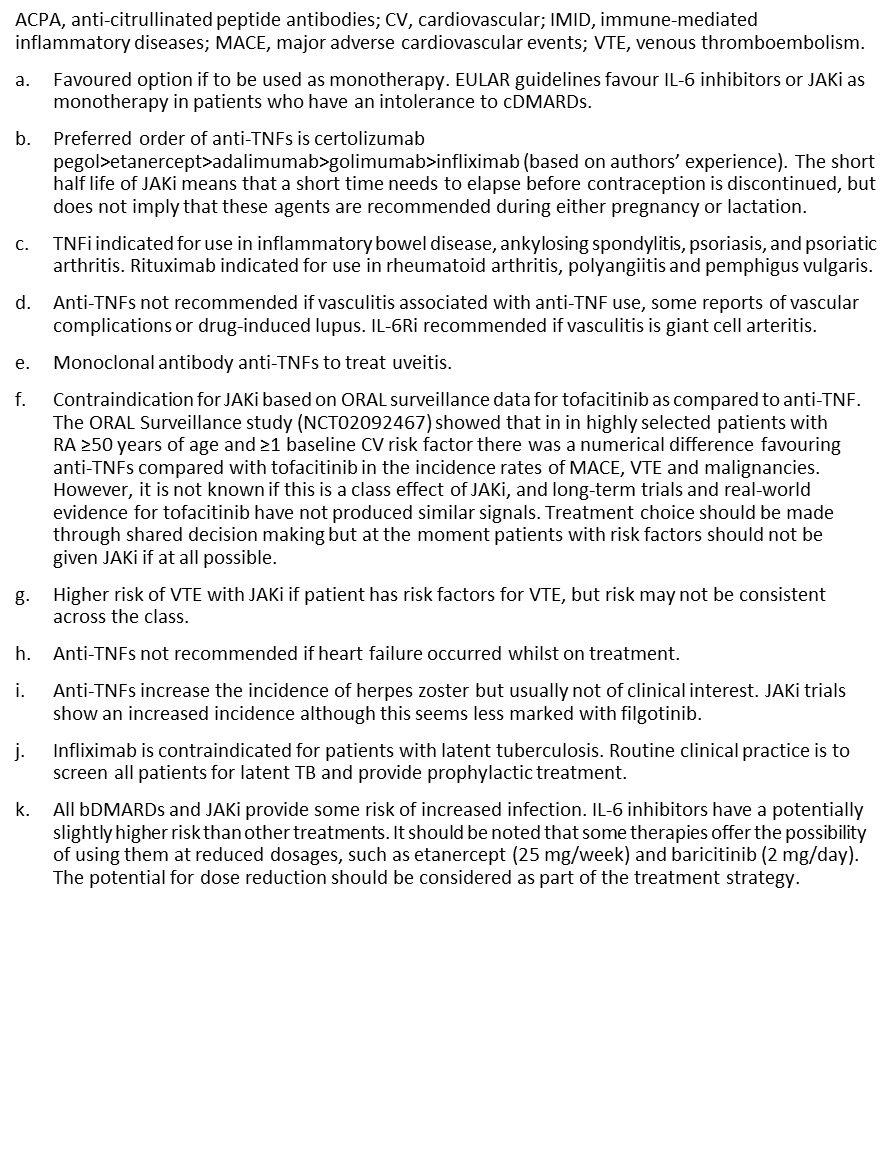
**

**References: Appendix Figure 1**

1. Smolen JS, Landewé RBM, Bijlsma JWJ, et al. EULAR recommendations for the management of rheumatoid arthritis with synthetic and biological disease-modifying antirheumatic drugs: 2019 update. *Ann Rheum Dis* 2020;79:685–99.
2. Flint J, Panchal S, Hurrell A, et al; BSR and BHPR Standards, Guidelines and Audit Working Group. BSR and BHPR guideline on prescribing drugs in pregnancy and breastfeeding-Part I: standard and biologic disease modifying anti-rheumatic drugs and corticosteroids. *Rheumatology (Oxford)* 2016;55:1693–7.
3. Götestam Skorpen C, Hoeltzenbein M, Tincani A, et al. The EULAR points to consider for use of antirheumatic drugs before pregnancy, and during pregnancy and lactation. *Ann Rheum Dis* 2016;75:795–810.
4. Murray KE, Moore L, O'Brien C, et al. Updated pharmacological management of rheumatoid arthritis for women before, during, and after pregnancy, reflecting recent guidelines. *Ir J Med Sci* 2019;188:169–72.
5. Chatzidionysiou K, Lie E, Nasonov E, et al. Highest clinical effectiveness of rituximab in autoantibody-positive patients with rheumatoid arthritis and in those for whom no more than one previous TNF antagonist has failed: pooled data from 10 European registries. *Ann Rheum Dis* 2011;70:1575–80.
6. Maniero RJ, Salgado E, Carmona L, Gomez-Reino JJ. Rheumatoid factor as predictor of response to abatacept, rituximab and tocilizumab in rheumatoid arthritis: Systematic review and meta-analysis. Semin Arthritis Rheum 2013;43:9-17
7. Lv Q, Yin Y, Li X, et al. The status of rheumatoid factor and anti-cyclic citrullinated peptide antibody are not associated with the effect of anti-TNFα agent treatment in patients with rheumatoid arthritis: a meta-analysis. *PLoS One*2014;9:e89442.
8. Cuppen BV, Welsing PM, Sprengers JJ, et al. Personalized biological treatment for rheumatoid arthritis: a systematic review with a focus on clinical applicability. *Rheumatology (Oxford)* 2016;55:826–39.
9. Kida D, Takahashi N, Kaneko A, et al. A retrospective analysis of the relationship between anti-cyclic citrullinated peptide antibody and the effectiveness of abatacept in rheumatoid arthritis patients. *Sci Rep* 2020;10:19717.
10. Alemao E, Postema R, Elbez Y, Mamane C, Finckh A. Presence of anti-cyclic citrullinated peptide antibodies is associated with better treatment response to abatacept but not to TNF inhibitors in patients with rheumatoid arthritis: a meta-analysis. Clin Exp Rheumatol 2020; 38: 455-66.
11. Takeuchi T. Treatment of rheumatoid arthritis with biological agents - as a typical and common immune-mediated inflammatory disease. *Proc Jpn Acad Ser B Phys Biol Sci* 2017;93:600–8.
12. Kaegi C, Wuest B, Schreiner J, et al. Systematic review of safety and efficacy of rituximab in treating immune-mediated disorders. *Front Immunol* 2019;10:1990.
13. Tocilizumab summary of product characteristics. <https://www.ema.europa.eu/en/documents/product-information/roactemra-epar-product-information_en.pdf>, accessed 22 March 2021.
14. Puéchal X, Gottenberg JE, Berthelot JM, et al; Investigators of the AutoImmunity Rituximab Registry. Rituximab therapy for systemic vasculitis associated with rheumatoid arthritis: Results from the AutoImmunity and Rituximab Registry. *Arthritis Care Res (Hoboken)* 2012;64:331–9.
15. Calderón-Goercke M, Castañeda S, Aldasoro V, et al. Tocilizumab in giant cell arteritis: differences between the GiACTA trial and a multicentre series of patients from the clinical practice. *Clin Exp Rheumatol* 2020;38 Suppl 124:112–9.
16. Leal I, Rodrigues FB, Sousa DC, et al. Efficacy and safety of intravitreal anti-tumour necrosis factor drugs in adults with non-infectious uveitis - a systematic review. *Acta Ophthalmol* 2018;96:e665–75.
17. Ming S, Xie K, He H, et al. Efficacy and safety of adalimumab in the treatment of non-infectious uveitis: a meta-analysis and systematic review. *Drug Des Devel Ther* 2018;12:2005–16.
18. Goto H, Zako M, Namba K, et al. Adalimumab in active and inactive, non-infectious uveitis: Global results from the VISUAL I and VISUAL II trials. *Ocul Immunol Inflamm* 2019;27:40–50.
19. Leal I, Rodrigues FB, Sousa DC, et al. Anti-TNF drugs for chronic uveitis in adults - A systematic review and meta-analysis of randomized controlled trials. *Front Med (Lausanne)* 2019;6:104.
20. Thanigaimani S, Phie J, Krishna SM, Moxon J, Golledge J. Effect of disease modifying anti‑rheumatic drugs on major cardiovascular events: a meta‑analysis of randomized controlled trials. Sci Rep 2021;11:6627.
21. Pfizer press release. Pfizer shares co-primary endpoint results from post-marketing required safety study of Xeljanz® (tofacitinib) in subjects with rheumatoid arthritis (RA). Available from: <https://www.pfizer.com/news/press-release/press-release-detail/pfizer-shares-co-primary-endpoint-results-post-marketing>, accessed 17th July 2021
22. Singh S, Fumery M, Singh AG, et al. Comparative risk of cardiovascular events with biologic and synthetic disease-modifying antirheumatic drugs in patients with rheumatoid arthritis: A systematic review and meta-analysis. *Arthritis Care Res (Hoboken)* 2020;72:561–76.
23. Giles JT, Sattar N, Gabriel S, et al. Cardiovascular safety of tocilizumab versus etanercept in rheumatoid arthritis: A randomized controlled trial. *Arthritis Rheumatol* 2020;72:31–40.
24. Taylor PC, Weinblatt ME, Burmester GR, et al. Cardiovascular safety during treatment with baricitinib in rheumatoid arthritis. *Arthritis Rheumatol* 2019;71:1042–5.
25. Roubille C, Richer V, Starnino T, et al. The effects of tumour necrosis factor inhibitors, methotrexate, non-steroidal anti-inflammatory drugs and corticosteroids on cardiovascular events in rheumatoid arthritis, psoriasis and psoriatic arthritis: a systematic review and meta-analysis. *Ann Rheum Dis* 2015;74:480–9.
26. Ozen G, Pedro S, Michaud K. The risk of cardiovascular events associated with disease-modifying antirheumatic drugs in rheumatoid arthritis. J Rheumatol 2021;48:648–55.
27. Charles-Schoeman C, Buch M, Dougados M, et al. Risk factors for major adverse cardiovascular events in patients aged ≥50 years with RA and ≥1 additional cardiovascular risk factor: results from a Phase 3b/4 randomized safety study of tofacitinib vs TNF inhibitors. Arthritis Rheumatol 2021;73(suppl 10): abstract 0958.
28. 28. Ytterberg SR, Bhatt DL, Mikuls TR, et al. Cardiovascular and cancer risk with tofacitinib in rheumatoid arthritis. *N Engl J Med*. 2022; 386:316-326
29. Curtis J, Yamaoka K, Chen Y-H, et al. Malignancies in patients aged ≥50 years with RA and ≥1 additional cardiovascular risk factor: results from a Phase 3b/4 randomized safety study of tofacitinib vs TNF inhibitors. Arthritis Rheumatol 2021;73(suppl 10): abstract 1940.
30. Charles-Schoeman, Fleischmann R, Mysler E, et al. The risk of venous thromboembolic events in patients with RA aged ≥50 years with ≥1 cardiovascular risk factor: results from a Phase 3b/4 randomixed safety study of tofacitinib vs TNF inhibitors. Arthritis Rheumatol 2021;73(suppl 10): abstract 1941.
31. Low AS, Symmons DP, Lunt M, et al. British Society for Rheumatology Biologics Register for Rheumatoid Arthritis (BSRBR-RA) and the BSRBR Control Centre Consortium. Relationship between exposure to tumour necrosis factor inhibitor therapy and incidence and severity of myocardial infarction in patients with rheumatoid arthritis. *Ann Rheum Dis* 2017;76:654–60.
32. Xeljanz SmPC, January 2021. Available from: <https://www.ema.europa.eu/en/documents/product-information/xeljanz-epar-product-information_en.pdf>, accessed 17th July 2021.
33. Sepriano A, Kerschbaumer A, Smolen JS, et al. Safety of synthetic and biological DMARDs: a systematic literature review informing the 2019 update of the EULAR recommendations for the management of rheumatoid arthritis. *Ann Rheum Dis* 2020;79:760–70.
34. Fraenkel L, Bathon JM, England BR, et al. 2021 American College of Rheumatology guideline for the treatment of rheumatoid arthritis. Arthritis Care Res 2021;73:924–939.
35. Isaacs JD, Harari O, Kobold U, et al. Effect of tocilizumab on haematological markers implicates interleukin-6 signalling in the anaemia of rheumatoid arthritis. *Arthritis Res Ther* 2013;15:R204.
36. Song SN, Iwahashi M, Tomosugi N, et al. Comparative evaluation of the effects of treatment with tocilizumab and TNF-α inhibitors on serum hepcidin, anemia response and disease activity in rheumatoid arthritis patients. *Arthritis Res Ther* 2013;15:R141.
37. Corrado A, Di Bello V, d'Onofrio F, et al. Anti-TNF-α effects on anemia in rheumatoid and psoriatic arthritis. *Int J Immunopathol Pharmacol* 2017;30:302–7.
38. Paul SK, Montvida O, Best JH, et al. Effectiveness of biologic and non-biologic antirheumatic drugs on anaemia markers in 153,788 patients with rheumatoid arthritis: New evidence from real-world data. *Semin Arthritis Rheum* 2018;47:478–84.
39. Pappas DA, St John G, Etzel CJ, et al. Comparative effectiveness of first-line tumour necrosis factor inhibitor versus non-tumour necrosis factor inhibitor biologics and targeted synthetic agents in patients with rheumatoid arthritis: results from a large US registry study. *Ann Rheum Dis* 2021;80:96–102.
40. Burmester GR, Hagino O, Dong Q, et al. Unique changes in hemoglobin with sarilumab versus adalimumab are independent of better disease control in patients with rheumatoid arthritis (RA). *Arthritis Rheumatol* 2018;70(suppl 10): Abstract 1528.
41. Genovese MC, Burmester GR, Hagino O, et al. Interleukin-6 receptor blockade or TNFα inhibition for reducing glycaemia in patients with RA and diabetes: post hoc analyses of three randomised, controlled trials. *Arthritis Res Ther* 2020;22:206.
42. Otsuka Y, Kiyohara C, Kashiwado Y, et al. Effects of tumor necrosis factor inhibitors and tocilizumab on the glycosylated hemoglobin levels in patients with rheumatoid arthritis; an observational study. *PLoS One* 2018;13:e0196368.
43. Chen SK, Lee H, Jin Y, Liu J, Kim SC. Use of biologic or targeted-synthetic disease-modifying anti-rheumatic drugs and risk of diabetes treatment intensification in patients with rheumatoid arthritis and diabetes mellitus. Rheumatol Adv Pract 2020;4:rkaa027.
44. Paul SK, Montvida O, Best JH, Gale S, Petho-Schramm A, Sarsour K. Association of biological antirheumatic therapy with risk for type 2 diabetes: a retrospective cohort study in incident rheumatoid arthritis. BMJ Open 2021;11:e042246.
45. Fleischmann R, Genovese MC, Lin Y, et al. Long-term safety of sarilumab in rheumatoid arthritis: an integrated analysis with up to 7 years' follow-up. *Rheumatology (Oxford)* 2020;59:292–302.
46. Harrington R, Al Nokhatha SA, Conway R. JAK inhibitors in rheumatoid arthritis: An evidence-based review on the emerging clinical data. *J Inflamm Res* 2020;13:519–31.
47. Lee YH, Song GG. Relative efficacy and safety of tofacitinib, baricitinib, upadacitinib, and filgotinib in comparison to adalimumab in patients with active rheumatoid arthritis. *Z Rheumatol* 2020;79:785–96.
48. Simon TA, Dong L, Winthrop KL. Risk of opportunistic infections in patients with rheumatoid arthritis initiating abatacept: cumulative clinical trial data. *Arthritis Res Ther* 2021a;23:17.
49. Liao TL, Chen YM, Liu HJ, Chen DY. Risk and severity of herpes zoster in patients with rheumatoid arthritis receiving different immunosuppressive medications: a case–control study in Asia. BMJ Open 2017;7:e014032.
50. Souto A, Maneiro JR, Salgado E, Carmona L, Gomez-Reino JJ. Risk of tuberculosis in patients with chronic immune-mediated inflammatory diseases treated with biologics and tofacitinib: a systematic review and meta-analysis of randomized controlled trials and long-term extension studies. *Rheumatology (Oxford)* 2014;53:1872–85.
51. Ai JW, Zhang S, Ruan QL, et al. The risk of tuberculosis in patients with rheumatoid arthritis treated with tumor necrosis factor-α antagonist: A metaanalysis of both randomized controlled trials and Registry/Cohort studies. *J Rheumatol* 2015;42:2229–37.
52. Riley TR, George MD. Risk for infections with glucocorticoids and DMARDs in patients with rheumatoid arthritis. RMD Open 2021;7:e001235.
53. Aaltonen KJ, Joensuu JT, Virkki L, et al. Rates of serious infections and malignancies among patients with rheumatoid arthritis receiving either tumor necrosis factor inhibitor or rituximab therapy. *J Rheumatol* 2015;42:372–8.
54. Singh JA, Cameron C, Noorbaloochi S, et al. Risk of serious infection in biological treatment of patients with rheumatoid arthritis: a systematic review and meta-analysis. *Lancet* 2015;386:258–65.
55. Simon TA, Boers M, Hochberg M, et al. Comparative risk of malignancies and infections in patients with rheumatoid arthritis initiating abatacept versus other biologics: a multi-database real-world study. *Arthritis Res Ther* 2019;21:228.
56. Lopez-Olivo MA, Tayar JH, Martinez-Lopez JA, et al. Risk of malignancies in patients with rheumatoid arthritis treated with biologic therapy: a meta-analysis. *JAMA* 2012;308:898–908.
57. Maneiro JR, Souto A, Gomez-Reino JJ. Risks of malignancies related to tofacitinib and biological drugs in rheumatoid arthritis: Systematic review, meta-analysis, and network meta-analysis. *Semin Arthritis Rheum* 2017;47:149–56.
58. de Germay S, Bagheri H, Despas F, Rousseau V, Montastruc F. Abatacept in rheumatoid arthritis and the risk of cancer: a world observational post-marketing study. *Rheumatology (Oxford)* 2020;59:2360–7.
59. Lopez-Olivo MA, Colmegna I, Karpes Matusevich AR, et al. Systematic review of recommendations on the use of disease-modifying antirheumatic drugs in patients with rheumatoid arthritis and cancer. *Arthritis Care Res (Hoboken)* 2020;72:309–18.
60. Xie W, Yang X, Huang H, Gao D, Ji L, Zhang Z. Risk of malignancy with non-TNFi biologic or tofacitinib therapy in rheumatoid arthritis: A meta-analysis of observational studies. *Semin Arthritis Rheum* 2020a;50:930–7.
61. Xie W, Xiao S, Huang Y, et al. A meta-analysis of biologic therapies on risk of new or recurrent cancer in patients with rheumatoid arthritis and a prior malignancy. *Rheumatology (Oxford)* 2020b;59:930–9.
62. Pfizer. Press release. Pfizer shares co-primary endpoint results from post-marketing required safety study of Xeljanz^®^ (tofacitinib) in subjects with rheumatoid arthritis (RA). <https://www.pfizer.com/news/press-release/press-release-detail/pfizer-shares-co-primary-endpoint-results-post-marketing>, accessed 30 May 2021.
63. Carrasco Cubero C, Chamizo Carmona E, Vela Casasempere P. Systematic review of the impact of drugs on diffuse interstitial lung disease associated with rheumatoid arthritis. *Reumatol Clin (Engl Ed)* 2021:17:504–13.
64. Fernández-Díaz C, Castañeda S, Melero-González RB, et al. Abatacept in interstitial lung disease associated with rheumatoid arthritis: national multicenter study of 263 patients. *Rheumatology (Oxford)* 2020;59:3906–16.
65. Narváez J, Robles-Pérez A, Molina-Molina M, et al. Real-world clinical effectiveness of rituximab rescue therapy in patients with progressive rheumatoid arthritis-related interstitial lung disease. *Semin Arthritis Rheum* 2020;50:902–10.
66. Kelly CA, Nisoar M, Arthanari S, et al. Rheumatoid arthritis related interstitial lung disease – improving outcomes over 25 years: a large multicentre UK study. *Rheumatology (Oxford)* 2020;60:1882–90.
67. Maini RN, Taylor PC, Szechinski J, et al; CHARISMA Study Group. Double-blind randomized controlled clinical trial of the interleukin-6 receptor antagonist, tocilizumab, in European patients with rheumatoid arthritis who had an incomplete response to methotrexate. *Arthritis Rheum* 2006;54:2817–29.
68. Yazici Y, Curtis JR, Ince A, et al. Efficacy of tocilizumab in patients with moderate to severe active rheumatoid arthritis and a previous inadequate response to disease-modifying antirheumatic drugs: the ROSE study. *Ann Rheum Dis* 2012;71:198–205.
69. Keystone EC, Taylor PC, Tanaka Y, et al. Patient-reported outcomes from a phase 3 study of baricitinib versus placebo or adalimumab in rheumatoid arthritis: secondary analyses from the RA-BEAM study. Ann Rheum *Dis* 2017;76:1853–61.
70. Smolen JS, Kremer JM, Gaich CL, et al. Patient-reported outcomes from a randomised phase III study of baricitinib in patients with rheumatoid arthritis and an inadequate response to biological agents (RA-BEACON). *Ann Rheum Dis* 2017b;76:694–700.
71. Strand V, Schiff M, Tundia N, et al. Effects of upadacitinib on patient-reported outcomes: results from SELECT-BEYOND, a phase 3 randomized trial in patients with rheumatoid arthritis and inadequate responses to biologic disease-modifying antirheumatic drugs. *Arthritis Res Ther* 2019;21:263.
72. Fautrel B, Zhu B, Taylor PC, et al. Comparative effectiveness of improvement in pain and physical function for baricitinib versus adalimumab, tocilizumab and tofacitinib monotherapies in rheumatoid arthritis patients who are naïve to treatment with biologic or conventional synthetic disease-modifying antirheumatic drugs: a matching-adjusted indirect comparison. *RMD Open* 2020;6:e001131.
73. Taylor PC, Lee YC, Fleischmann R, et al. Achieving pain control in rheumatoid arthritis with baricitinib or adalimumab plus methotrexate: Results from the RA-BEAM Trial. *J Clin Med* 2019;8:831.
74. Gout T, Ostör AJ, Nisar MK. Lower gastrointestinal perforation in rheumatoid arthritis patients treated with conventional DMARDs or tocilizumab: a systematic literature review. *Clin Rheumatol* 2011;30:1471–4.
75. Cohen SB, Tanaka Y, Mariette X, et al. Long-term safety of tofacitinib for the treatment of rheumatoid arthritis up to 8.5 years: integrated analysis of data from the global clinical trials. *Ann Rheum Dis* 2017;76:1253–62.
76. Strangfeld A, Richter A, Siegmund B, et al. Risk for lower intestinal perforations in patients with rheumatoid arthritis treated with tocilizumab in comparison to treatment with other biologic or conventional synthetic DMARDs. *Ann Rheum Dis* 2017;76:504–10.
77. Cohen SB, van Vollenhoven RF, Winthrop KL, et al. Safety profile of upadacitinib in rheumatoid arthritis: integrated analysis from the SELECT phase III clinical programme. *Ann Rheum Dis* 2020a:annrheumdis-2020-218510.
78. Watson P, Simler N, Screaton N, Lillicrap M. Management of accelerated pulmonary nodulosis following etanercept therapy in a patient with rheumatoid arthritis. *Rheumatology (Oxford)* 2008;47:928–9.
79. van Ede A, den Broeder A, Wagenaar M, van Riel P, Creemers MC. Etanercept-related extensive pulmonary nodulosis in a patient with rheumatoid arthritis. *J Rheumatol* 2007;34:1590–2.
80. Verschueren K, Van Essche E, Verschueren P, Taelman V, Westhovens R. Development of sarcoidosis in etanercept-treated rheumatoid arthritis patients. *Clin Rheumatol* 2007;26:1969–71.
81. Glace B, Gottenberg J-E, Mariette X, et al. Efficacy of rituximab in the treatment of pulmonary rheumatoid nodules: findings in 10 patients from the French AutoImmunity and Rituximab/Rheumatoid Arthritis registry (AIR/PR registry). Ann Rheum Dis 2012;71:1429–31.
82. Braun MG, Wagener P. Regression von peripheren und pulmonalen Rheumaknoten unter Rituximab-Therapie. Z Rheumatol 2013;72:166–71.
83. De Stefano R, Frati E, Nargi F, Menza L. Efficacy of rituximab on pulmonary nodulosis occurring or increasing in patients with rheumatoid arthritis during anti-TNF-α therapy. Clin Exp Rheumatol 2011;29:752–3.
84. Bernatsky S, Renoux C, Suissa S. Demyelinating events in rheumatoid arthritis after drug exposures. Ann Rheum Dis 2010;69:1691–3.
85. Seror R, Richez C, Sordet C, et al. Pattern of demyelination occurring during anti-TNF-α therapy: a French national survey. Rheumatology 2013;52:868–74.
86. Taylor TRP, Galloway J, Davies R, Hyrich K, Dobson R. Demyelinating events following initiation of anti-TNFα therapy in the British Society for Rheumatology Biologics Registry in Rheumatoid Arthritis. Neurol Neuroimmunol Neuroinflamm 2021;8:e992.
