# Supplementary material for: Managing inadequate response to initial anti-TNF therapy in rheumatoid arthritis: optimising treatment outcomes
Source: Ther Adv Musculoskelet Dis. 2022 Aug 16;14:1759720X221114101. doi: 10.1177/1759720X221114101 (PMC9386864; doi:10.1177/1759720X221114101)
Supplement: sj-pptx-1-tab-10.1177_1759720X221114101 – Supplemental material for Managing inadequate response to initial anti-TNF therapy in rheumatoid arthritis: optimising treatment outcomes [file sj-pptx-1-tab-10.1177_1759720X221114101.pptx]

## Slide 1
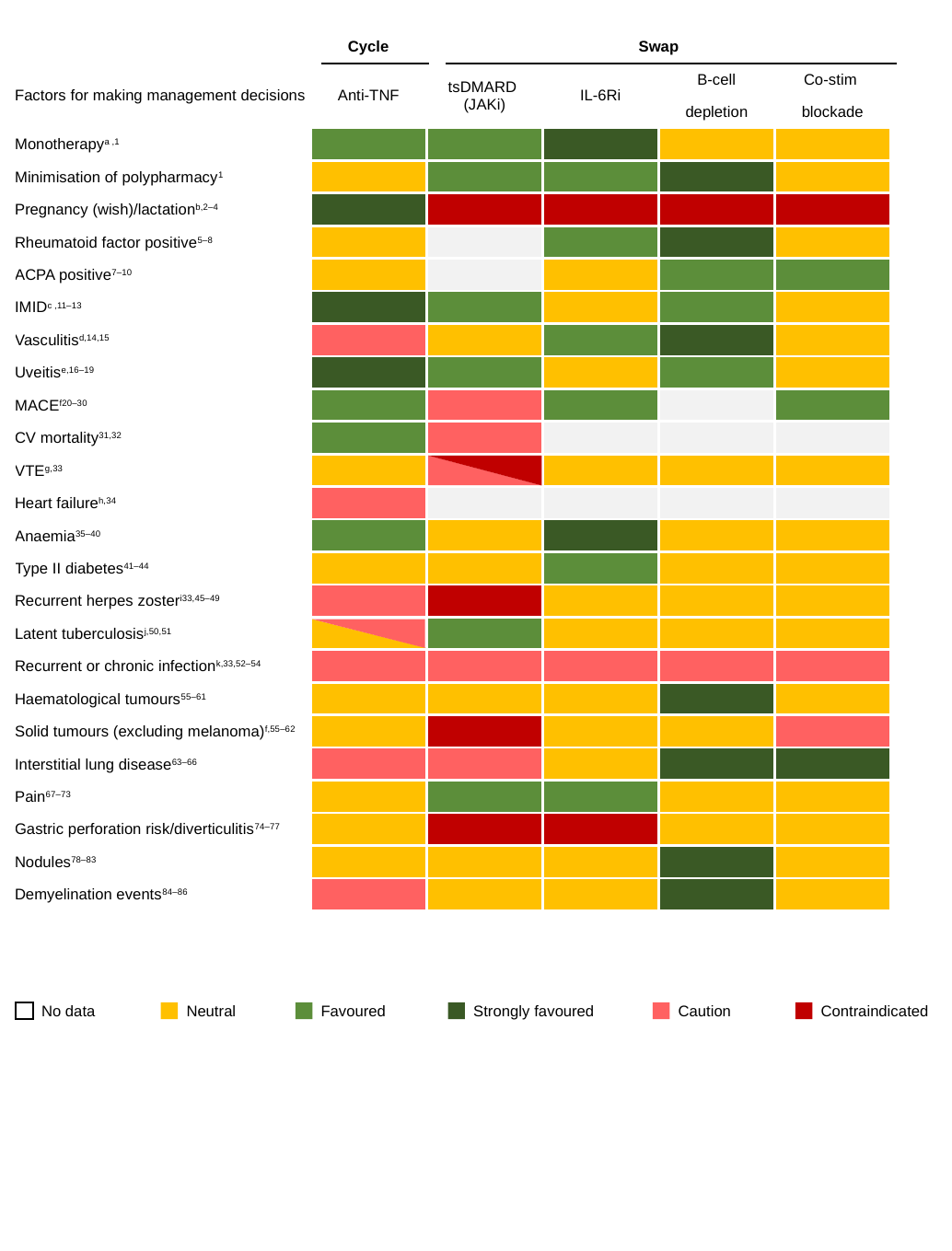

| | Cycle | Swap | | | |
| --- | --- | --- | --- | --- | --- |
| Factors for making management decisions | Anti-TNF | tsDMARD (JAKi) | IL-6Ri | B-cell | Co-stim |
| | | | | depletion | blockade |
| Monotherapya ,1 | | | | | |
| Minimisation of polypharmacy1 | | | | | |
| Pregnancy (wish)/lactationb,2–4 | | | | | |
| Rheumatoid factor positive5–8 | | | | | |
| ACPA positive7–10 | | | | | |
| IMIDc ,11–13 | | | | | |
| Vasculitisd,14,15 | | | | | |
| Uveitise,16–19 | | | | | |
| MACEf20–30 | | | | | |
| CV mortality31,32 | | | | | |
| VTEg,33 | | | | | |
| Heart failureh,34 | | | | | |
| Anaemia35–40 | | | | | |
| Type II diabetes41–44 | | | | | |
| Recurrent herpes zosteri33,45–49 | | | | | |
| Latent tuberculosisj,50,51 | | | | | |
| Recurrent or chronic infectionk,33,52–54 | | | | | |
| Haematological tumours55–61 | | | | | |
| Solid tumours (excluding melanoma)f,55–62 | | | | | |
| Interstitial lung disease63–66 | | | | | |
| Pain67–73 | | | | | |
| Gastric perforation risk/diverticulitis74–77 | | | | | |
| Nodules78–83 | | | | | |
| Demyelination events84–86 | | | | | |
No data
Neutral
Favoured
Strongly favoured
Caution
Contraindicated

## Slide 2
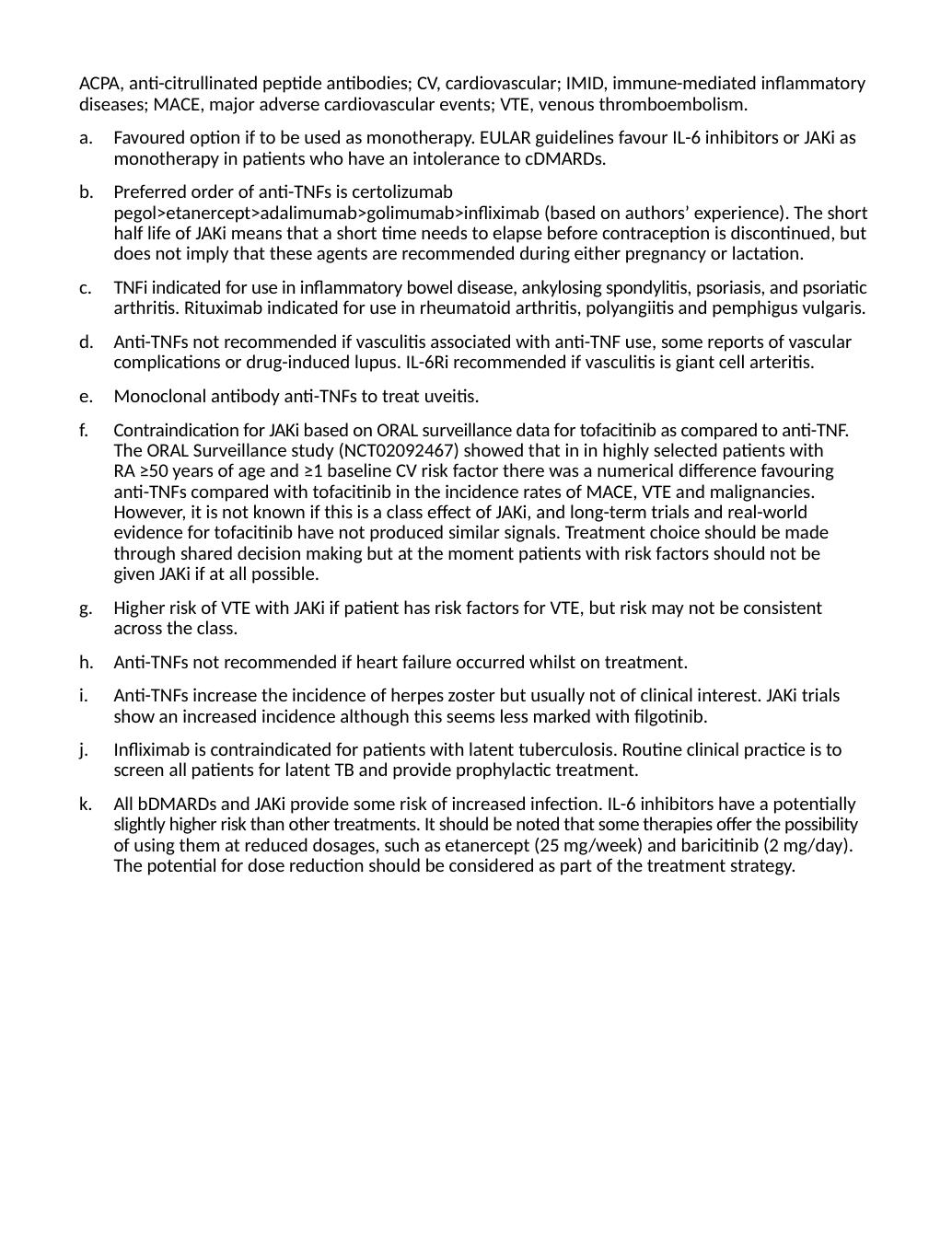

ACPA, anti-citrullinated peptide antibodies; CV, cardiovascular; IMID, immune-mediated inflammatory diseases; MACE, major adverse cardiovascular events; VTE, venous thromboembolism.
Favoured option if to be used as monotherapy. EULAR guidelines favour IL-6 inhibitors or JAKi as monotherapy in patients who have an intolerance to cDMARDs.
Preferred order of anti-TNFs is certolizumab pegol>etanercept>adalimumab>golimumab>infliximab (based on authors’ experience). The short half life of JAKi means that a short time needs to elapse before contraception is discontinued, but does not imply that these agents are recommended during either pregnancy or lactation.
TNFi indicated for use in inflammatory bowel disease, ankylosing spondylitis, psoriasis, and psoriatic arthritis. Rituximab indicated for use in rheumatoid arthritis, polyangiitis and pemphigus vulgaris.
Anti-TNFs not recommended if vasculitis associated with anti-TNF use, some reports of vascular complications or drug-induced lupus. IL-6Ri recommended if vasculitis is giant cell arteritis.
Monoclonal antibody anti-TNFs to treat uveitis.
Contraindication for JAKi based on ORAL surveillance data for tofacitinib as compared to anti-TNF. The ORAL Surveillance study (NCT02092467) showed that in in highly selected patients with
RA ≥50 years of age and ≥1 baseline CV risk factor there was a numerical difference favouring anti-TNFs compared with tofacitinib in the incidence rates of MACE, VTE and malignancies. However, it is not known if this is a class effect of JAKi, and long-term trials and real-world evidence for tofacitinib have not produced similar signals. Treatment choice should be made through shared decision making but at the moment patients with risk factors should not be
given JAKi if at all possible.
Higher risk of VTE with JAKi if patient has risk factors for VTE, but risk may not be consistent across the class.
Anti-TNFs not recommended if heart failure occurred whilst on treatment.
Anti-TNFs increase the incidence of herpes zoster but usually not of clinical interest. JAKi trials show an increased incidence although this seems less marked with filgotinib.
Infliximab is contraindicated for patients with latent tuberculosis. Routine clinical practice is to screen all patients for latent TB and provide prophylactic treatment.
All bDMARDs and JAKi provide some risk of increased infection. IL-6 inhibitors have a potentially slightly higher risk than other treatments. It should be noted that some therapies offer the possibility of using them at reduced dosages, such as etanercept (25 mg/week) and baricitinib (2 mg/day). The potential for dose reduction should be considered as part of the treatment strategy.
